# Biotransformation of Deoxynivalenol to the Novel Metabolite Deoxynivalenol-8,15-hemiketal-7-glucoside by the *Bacillus subtilis* Glycosyltransferase YjiC

**DOI:** 10.1021/acsomega.5c01301

**Published:** 2025-03-24

**Authors:** Shawn J. Hoogstra, Justin B. Renaud, David R. McMullin, Megan J. Kelman, Christopher P. Garnham, Mark W. Sumarah

**Affiliations:** 1 Agriculture and Agri-Food Canada, London Research and Development Centre, 1391 Sandford Street, London, ON N5V4T3, Canada; 2 Department of Chemistry, Carleton University, Ottawa, ON K1S 5B6, Canada

## Abstract

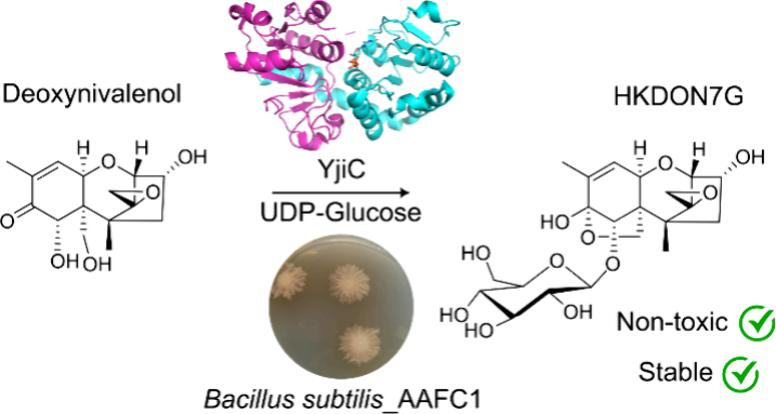

The mycotoxin deoxynivalenol (DON) is a chronic problem in cereals in temperate areas worldwide. Above regulatory levels, DON contamination can result in significant economic loss both to the primary producer and the feed industry in terms of increased costs. Here we report the enzymatic biotransformation of DON to a novel stable metabolite by a soil-borne strain of *Bacillus subtilis*. Proteomic analysis of activity-enriched protein fractions from this *B. subtilis* strain identified the glycosyltransferase YjiC as the enzyme responsible for the observed DON biotransformation. Liquid chromatography high-resolution tandem mass spectrometry and NMR spectroscopic analysis demonstrated that YjiC glycosylates DON at the 7-hydroxyl position, producing the novel metabolite DON-8,15-hemiketal-7-glucoside (HKDON7G). In toxicity experiments, duckweed exposed to 20 μM HKDON7G showed no phytotoxicity when compared to DON. Stability testing of HKDON7G demonstrated that it is significantly more resistant to enzymatic and microbial hydrolysis compared to DON-3-glucoside. This study is the first to report a chemical modification to the 7-hydroxyl position of DON and presents a novel mechanism for the detoxification of DON-contaminated food and feed.

## Introduction

**Figure 1:**
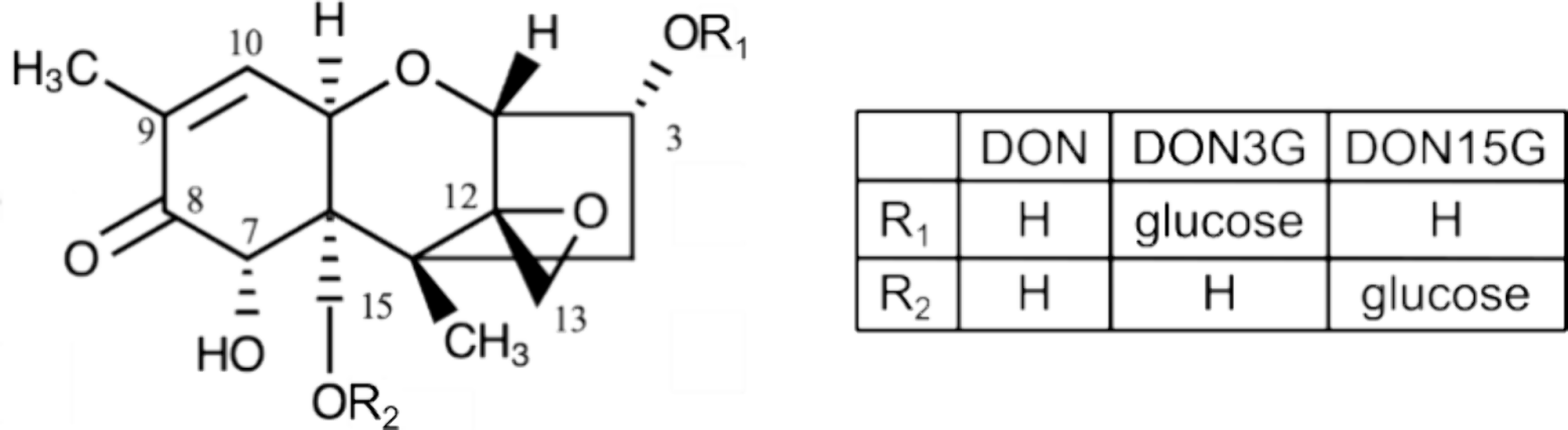
Chemical structure of deoxynivalenol (DON) and its glycosylated derivatives DON-3-glucoside (DON3G) and the synthetically produced DON-15-glucoside (DON15G).

Mycotoxin contamination of crops is a global problem that is estimated to cost billions of dollars to the world economy in bad years.^1^ Deoxynivalenol (DON), also known as vomitoxin, is a mycotoxin produced by *Fusarium graminearum*, *F. culmorum* and *F. asiaticum.* These species cause Fusarium head blight in small grains and Gibberella ear rot in maize when there is rain at anthesis or silk emergence, respectively, under warm conditions.^2^ Cereals contaminated with DON above regulatory levels are deemed unsuitable for human consumption or animal feed. Swine are the most sensitive species to the effects of DON because of feed refusal and reduced weight gain.^3,4^ DON is a type B trichothecene mycotoxin, which is part of a large family of phytotoxic virulence factors that share a common tricyclic 12, 13-epoxytrichothec-9-ene scaffold (Figure 1). DON contains a ketone moiety at position 8, along with hydroxyl groups at positions 3, 7, and 15.

There are several effective mitigation strategies for limiting DON, including breeding of resistant genotypes, application of fungicides, grain sorting and testing.^5^ For those crops that are contaminated at concentrations above regulatory limits, detoxification is a potential method for remediating the commodity. There are a number of enzymes described in the literature that are capable of chemically altering and detoxifying most of the major mycotoxins including zearalenone,^6^ fumonisin,^7^ aflatoxin,^8^ and ochratoxin.^9^

DON has been reported to undergo various enzymatic modifications, including glycosylation, sulfation, acetylation, and glucuronidation, primarily at its 3- and 15-hydroxyl groups;^10−14^ oxidization and subsequent epimerization at the 3-hydroxyl group;^15,16^ isomerization at the C-7/C-8/C-9 positions;^17^ and reductive alkylation^18,19^ or glutathionylation^20^ at the C-12/C-13 epoxide moiety. These DON biotransformation products display reduced toxicity compared to the native compound. Unfortunately, many of these biotransformation products are difficult to produce because either 1) the enzyme(s) responsible for their production have yet to be discovered;^18,19^ 2) they require costly enzymatic cofactors;^16,21^ or 3) they simply temporarily mask the toxicity. Masking is particularly problematic for DON-3-glucoside (DON3G) (Figure 1), an abundant plant detoxification product that is readily converted back to DON by the mammalian microbiome upon ingestion.^22−24^ New tools capable of remediating DON-contaminated grains are needed to improve the economics of feed production in challenging years.

Agricultural microbiomes are potentially rich in organisms capable of DON biotransformation.^25,26^ Recently, two strains of *Bacillus* sp. with novel DON biotransformation activity were isolated from winter wheat fields in China.^27^*Bacillus* sp. HN117 and N22, which are both likely strains of *B. subtilis*, produced M-DOM, norDON E, and 9-hydroxymethyl DON lactone.^27^ The toxicity of the biotransformation products and the underlying enzymatic mechanism of their production is unknown. Another strain of *B. subtilis* (ASAG 216), was isolated from the intestine of a donkey.^28^ This bacterium was capable of significantly reducing DON concentrations in culture. However, as with *Bacillus* spp. HN117 and N22, the underlying biotransformation mechanisms are unknown.

We recently developed a liquid chromatography mass spectrometry (LC-MS)-based discovery pipeline to identify microbes and their corresponding enzymes capable of mycotoxin biotransformation.^29^ Here we employed this pipeline to screen for new enzymes capable of biotransforming DON. Soil samples were collected in the fall of 2018 from agricultural fields in southern Ontario, Canada. This year experienced one of the largest *F. graminearum* outbreaks in recent times with massive corn crop losses due to DON contamination above regulatory limits. This outbreak resulted in some fields being plowed into the ground. Here we describe the identification and characterization of a novel DON biotransformation metabolite catalyzed by a strain of *B. subtilis* isolated from these agricultural soils. This novel mechanism for DON detoxification warrants further investigation but shows promise as a new tool for the management of DON contaminated food and feed.

## Results and Discussion

### Application of the Discovery Pipeline for Microbes Capable of DON Biotransformation Activity

**Figure 2:**
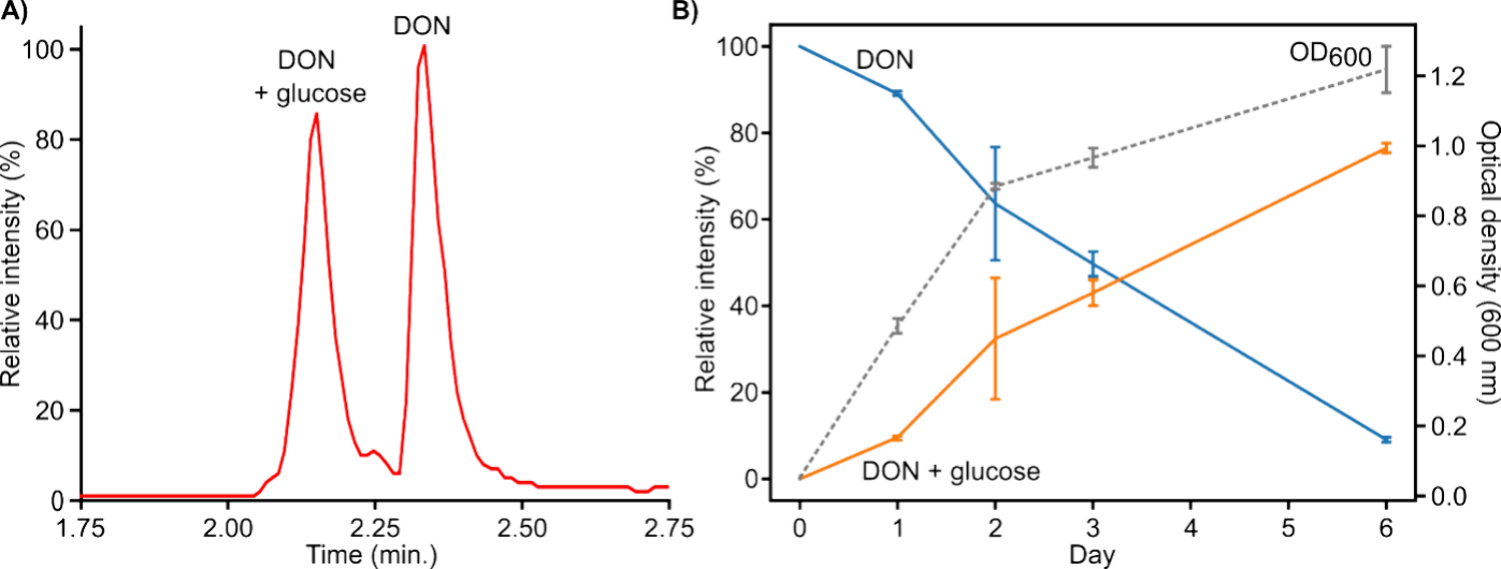
(A) Relative intensity levels from the combined negative mode ESI extracted ion chromatograms for DON (t_R_ = 2.33 min.) and the major DON biotransformation product (DON + glucose, *t*_R_ = 2.16 min.) after 72 h of bacterial growth. (B) Time-course of DON biotransformation shown by monitoring combined negative mode ESI extracted ion intensity levels of DON (blue line) and the DON + glucose biotransformation product (orange line). The dashed gray line represents the OD_600_ of the bacterial culture grown in YES media. Error bars represent standard deviation (*n* = 3).

We have developed a discovery pipeline that enables the screening of any material that contains a potentially complex mixed microbial community (e.g., soil, wastewater, animal excreta and gut contents) for mycotoxin degradation via LC-HRMS.^29,30^ Here we employed this pipeline to screen for DON biotransformation activity from soils sampled from several *Fusarium* contaminated agricultural fields in southern Ontario in the fall of 2018. Samples were incubated with DON, and during this screening, a mixed microbial culture incubated in a 1/100 dilution of YES media with the capacity to reduce the concentration of DON in solution was identified via LC-HRMS. To isolate the individual microbe(s) responsible for the observed DON biotransformation activity, a serial dilution of the culture on YES-Agar plates was performed. Individual colonies were picked and grown aerobically in YES media in the presence of DON, and one isolated bacterial colony with the ability to reduce the concentration of DON in liquid culture was identified and screened via nontargeted LC-HRMS (Figure 2). A major DON biotransformation product was observed in negative ionization mode with a [M - H]^−^ molecular ion at *m*/*z* 457.1715 that eluted earlier than DON (Figure 2A). The increased mass of the [M - H]^−^ ion at *m*/*z* 457.1715 compared to [DON + HCOO]^−^ corresponds to the addition of a single glucose moiety to the trichothecene scaffold (C_6_H_12_O_6_ -H_2_O; Δ = 162.0528 Da). Over a six-day growth period in YES media in the presence of DON, a gradual increase in the OD_600_ of the bacterial culture was observed, alongside a corresponding increase in the presence of the glycosylated biotransformation product, with a concomitant decrease in DON (Figure 2B).

### Biochemical Enrichment of Native DON-Biotransformation Activity

**Figure 3:**
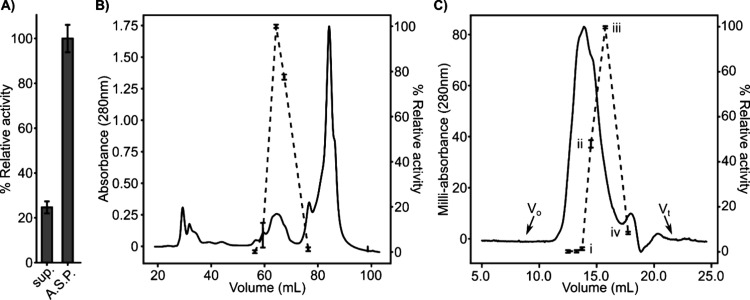
Enzymatic enrichment of DON-biotransformation activity from *B. subtilis*_AAFC1. (A) Relative DON biotransformation activity as determined via reverse phase LC-HRMS analysis of *B. subtilis*_AAFC1 culture pre- and postammonium sulfate precipitation. sup. = lysate supernatant post sonication and clarification of live culture; A.S.P. = resuspended protein pellet of a 60–90% (w:v) ammonium sulfate precipitation. Error bars represent standard deviation (*n* = 3). (B) Q-Sepharose enrichment of DON-biotransformation activity post ammonium sulfate precipitation. Black line represents absorbance at 280 nm, and the dashed line represents LC-HRMS intensity levels of the glycosylated DON product following incubation of individual fractions with DON and UDP-glucose (*n* ≥ 2). (C) Gel permeation chromatography enrichment of DON biotransformation activity post Q-Sepharose ion exchange. Both the black and dashed lines are the same as in panel B. *V*_o_ and *V_t_* represent the void and total volumes of the column, respectively. i-iv represent fractions subjected to LC-MS/MS proteomics analysis.

The 16S rRNA gene sequence of the isolated and sequenced bacterium was 99.94% identical to *Bacillus subtilis* subsp. subtilis strain 168 (NCBI Reference Sequence: NC_000964.3), with only one base pair difference between the two rRNA genes across their entire lengths. We therefore refer to our isolated strain as *B. subtilis*_AAFC1. To identify potential enzyme candidates from the microbe, DON biotransformation activity was biochemically enriched from liquid cultures of *B. subtlilis*_AAFC1. DON biotransformation was monitored after each enrichment step via LC-HRMS analysis of protein fractions administered DON and UDP-glucose. DON biotransformation activity was present primarily within the lysate supernatant of the culture (Figure 3A). A stepwise ammonium sulfate precipitation of the clarified lysate supernatant demonstrated DON biotransformation activity was significantly enriched in the precipitated pellet following a 60%–90% (w:v) fractionation (Figure 3A). This active fraction was next subjected to Q-Sepharose cation exchange chromatography (Figure 3B). DON biotransformation activity eluted discretely off the column at ca. 400 mM NaCl (Figure 3B). Active fractions were pooled and then separated via gel permeation chromatography, where DON biotransformation activity again eluted discretely, located primarily in the shoulder of the main elution peak. Fractions displaying activity following gel permeation enrichment (Figure 3C, i-iv) were subjected to trypsin digestion and LC-MS/MS proteomics analysis to identify candidate biotransformation enzymes (Table S1).

### Proteomics Analysis Identifies YjiC as Candidate for DON Biotransformation Activity

**Figure 4:**
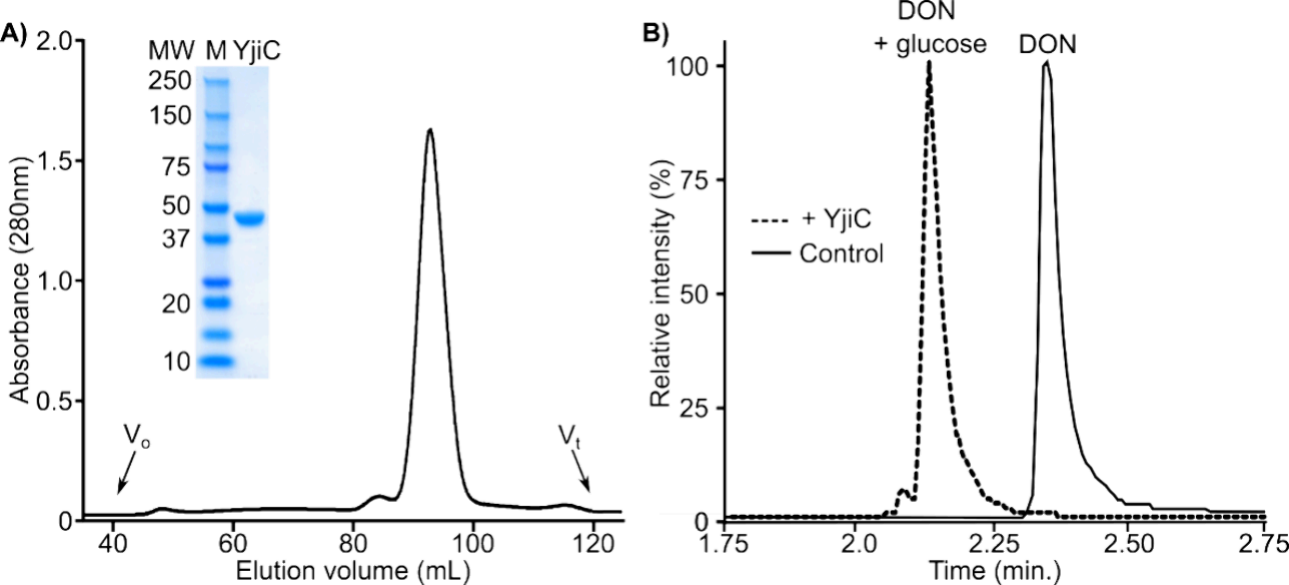
Purification and DON biotransformation activity of recombinant YjiC. (A) Gel permeation chromatogram of recombinant YjiC from *B. subtilis*_AAFC1. Black line represents absorbance at 280 nm. *V*_o_ = void volume; *V_t_* = total volume of the column. Inset is an SDS-PAGE analysis of pooled fractions of YjiC following chromatographic separation. M = protein markers. Numbers represent MW of each marker. (B) Relative intensity levels from the combined negative mode ESI extracted ion chromatograms are shown for DON (*t*_R_ = 2.33 min) and the YjiC-catalyzed DON biotransformation product (DON + glucose, *t*_R_ = 2.16 min). Black line represents control reaction with no YjiC added, while the hatched line represents the addition of YjiC.

The proteomics data were searched against the sequenced genome of *B. subtilis*_AAFC1. The top-ranked enzyme candidate from this analysis with the highest number of peptide spectral matches was to a putative UDP-glycosyltransferase, wherein 74 validated and unique peptides that corresponded to 72.7% sequence coverage of the entire enzyme were observed. BLASTp searches against the NCBI nonredundant protein database indicated the enzyme was highly similar to YjiC, a 392 amino acid UDP-glycosyltransferase, previously reported to be capable of glycosylating a diverse array of plant secondary metabolites and xenobiotics.^31−33^ This isoform of YjiC was 98.72% identical and contained only five amino acid substitutions (Asp97Glu, Arg148Lys, Lys210Gln, Ser212Gly, and Ser359Thr) compared to its homologue found in *B. subtilis* strain 168 (NCBI ref. Seq NP_389104.1). The newly identified homologue of YjiC was synthesized and cloned as a 6X-His-tagged N-terminal MBP fusion protein and was overexpressed in *E. coli*. Following TEV protease cleavage, the 44.16-kDa recombinant protein was purified to homogeneity as witnessed via SDS-PAGE analysis of pooled fractions following gel permeation chromatography (Figure 4A). Following purification, YjiC was incubated with DON and UDP-glucose and the reaction was monitored by LC-HRMS (Figure 4B). YjiC generated the identical monoglycosylated DON product with a t_R_ of 2.16 min that was produced by cultures of *B. subtilis*_AAFC1. Minor amounts of an additional DON mono- and diglucoside product were also observed (data not shown).

### Structural Elucidation of the YjiC-Catalyzed DON Biotransformation Product

**Figure 5:**
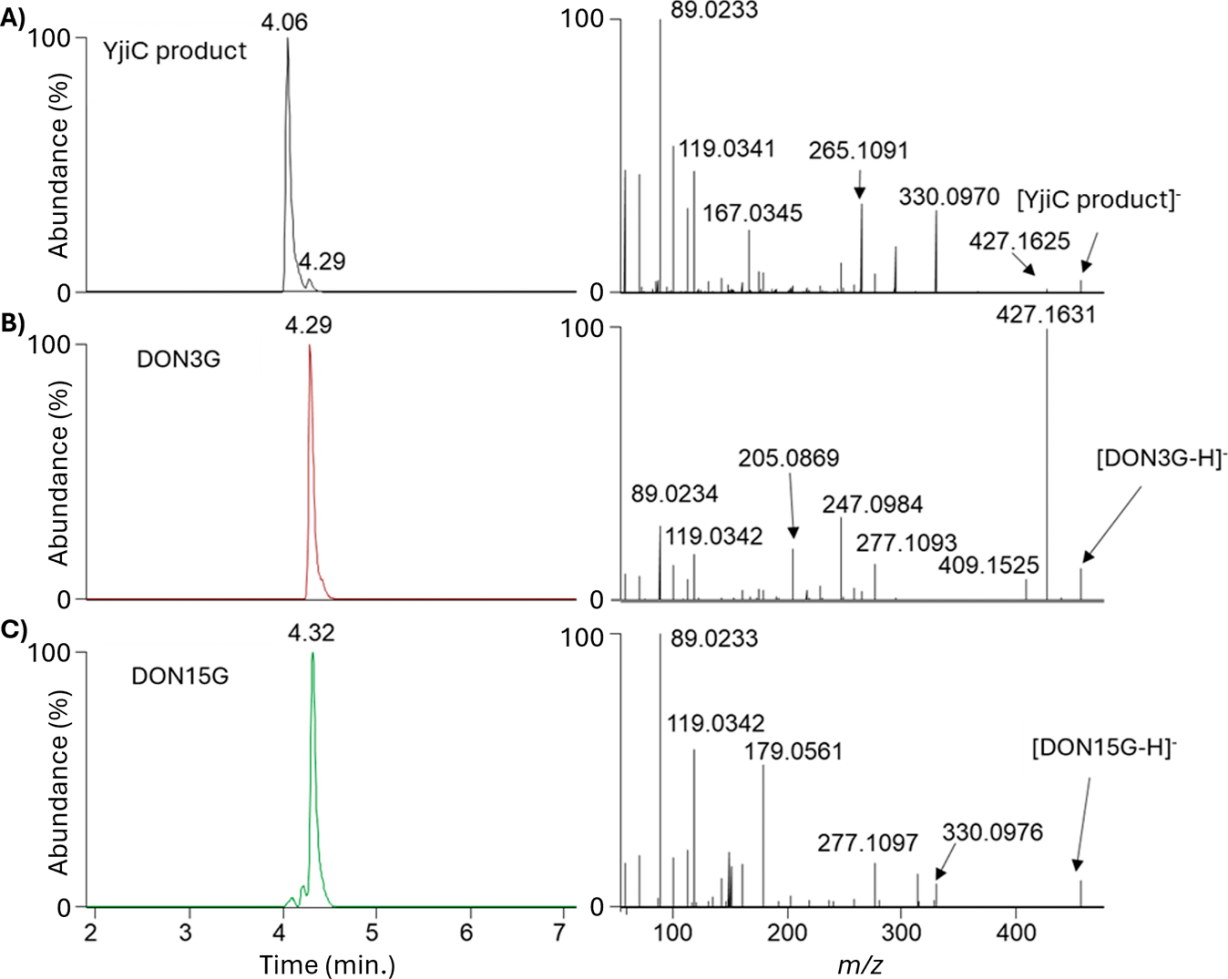
LC-MS/MS spectra showing the % abundance of the main YjiC biotransformation product (A), DON3G (B), and DON15G (C).

The YjiC-catalyzed DON biotransformation product was compared to commercially obtained DON3G (Figure 1) by LC-MS/MS using a tailored HPLC gradient for further characterization of its structure (Figure 5). DON3G eluted at 4.29 min in this method, whereas the major YjiC-derived product was more polar, eluting at 4.06 min. Additionally, the main adduct for DON3G is formate ([M+HCOO]^−^) whereas the YjiC product appears to be more acidic and is mainly observed as the deprotonated adduct ([M-H]^−^). This difference in retention times and preferred adduct provides evidence for the structural uniqueness of the YjiC-catalyzed biotransformation product. To determine if YjiC potentially glycosylates DON at the 15-hydroxyl position, a standard of DON15G (Figure 1) was produced synthetically using the reactant 3-acetyl-DON (3ADON) as the glycosyl acceptor in a Koenigs-Knorr reaction. Interestingly, DON15G also had a distinct t_R_ (4.32 min) as compared to the YjiC product, indicating that YjiC is producing neither DON3G nor DON15G.

Interpretation of the MS/MS product ion spectra of DON3G, DON15G, and the YjiC biotransformation product revealed key differences corresponding to their molecular structures (Figure 5). Specifically, the diagnostic product ion at the theoretical *m*/*z* 427.1610, which is generated by the neutral loss of the oxygenated methylene, as CH_2_O, at position C-15. This is the major product ion observed in the MS/MS spectrum of DON3G. As expected, analysis of the MS/MS spectrum of the synthetically produced DON15G showed that glycosylation at position 15 prevents the CH_2_O neutral loss, evidenced by the absence of this product ion (Figure 5C). In the MS/MS spectrum for the YjiC biotransformation product, this diagnostic product ion was observed, albeit at much lower intensities than DON3G. Along with the difference in t_R_ between DON15G and the fact that a small product ion at *m/*z 427.1625 is observed demonstrates that glycosylation is not occurring at the C-15 hydroxyl. Conversely, the lower intensity of this diagnostic product ion suggests a more complex biotransformation mechanism involving the hydroxyl moiety at position C-15 is happening.

**Figure 6:**
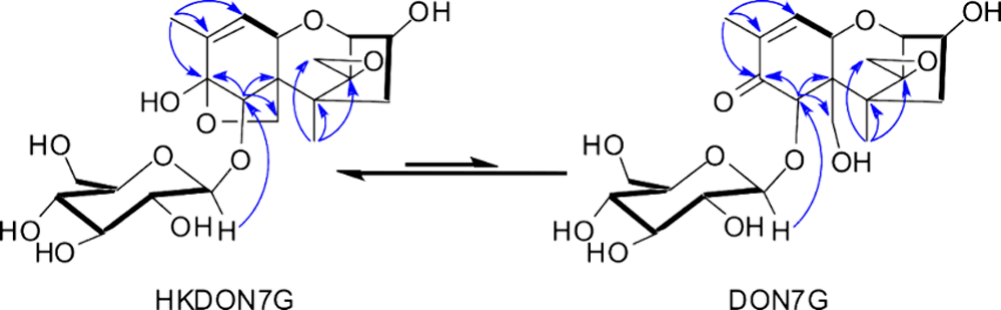
Chemical structures of the tautomers 8,15 hemiketal-7-*O*-β-d-glucoside (HKDON7G) and deoxynivalenol-7-*O*-β-d-glucoside (DON7G) generated by the YjiC-catalyzed reaction of DON and UDP-glucose. Observed COSY (bold black) and key HMBC correlations (blue arrows) used to determine the planar structure of the DON YjiC biotransformation product structural isomers in CD_3_OD are shown.

Flash chromatography was used to purify the biotransformation product from the YjiC-catalyzed reaction of DON and UDP-glucose for structural characterization. NMR spectra (Figures S1–S5) were recorded in CD_3_OD to enable comparisons to other trichothecene glucosides and related compounds.^14,34,35^ Interpretation of the homonuclear (^1^H and ^13^C, COSY) and heteronuclear (HSQC and HMBC) NMR spectra revealed that the biotransformation product exists in an equilibrium as two structural isomers, each consisting of a trichothecene aglycone and a single glucopyranosyl spin system, consistent with the MS/MS data (Table 1; Figures 5 and 6). Integration of the ^1^H NMR signals showed that the tautomers exist in a 3:2 molar ratio in CD_3_OD. Observed HMBC correlations and the chemical shifts of the C-3 and C-15 signals indicated neither trichothecene tautomer was O-glycosylated at these positions, further supporting the MS/MS data. The NMR data for the major isomer showed similarity with the DON hemiketal tautomer.^14,36^ Accordingly, the olefinic signal at δ 5.44 (dd, 4.1, 1.8; H-10) was approximately 1 ppm more shielded compared to the DON enone structural isomer, no carbonyl signal was observed, and HMBC correlations were observed from δ 4.16 (s; H-7) and δ 1.77 (bs; H-16) to a ketal carbon at δ 106.5 (C-8) instead of a carbonyl. Further, a smaller geminal coupling constant for the H-15 diastereotopic methylene protons at δ 4.16 (d, 8.5) and δ 3.37 (d, 8.5) was observed. The geminal H-15 coupling constant for the DON enone tautomer is reported in the 10–13 Hz range. Together, these data confirm the new DON metabolite exists as the energetically less favorable hemiketal tautomer. The β*-*configuration of the glycosidic linkage was assigned based on the anomeric H-1′ coupling constant, δ 4.73 (d, 8.0), and the C-1’ chemical shift at δ 104.8. The larger H1′_ax_–H2′_ax_ bond angle for the β*-*anomer results in a larger *J*_1′_,_2′_ coupling constant. For an α-anomer, a smaller characteristic *J*_1’_,_2’_ coupling constant in the 1–4 Hz range would be expected due to a smaller H1′_eq_–H2′_ax_ dihedral angle. The chemical shift of C-1′ is also characteristic of DON β-glucosides, in the 100–104 ppm range, whereas α-glucosides would have a lower chemical shift in the 95–100 ppm range.^34^ HMBC correlations were observed from the anomeric H-1′ resonance and one H-15 methylene signal to the deshielded oxygenated methine at δ 83.6 (C-7) connecting the hemiketal DON and glucopyranosyl spin systems. The NMR and MS/MS data indicate that the structure of the major isomer for the YjiC-catalyzed DON biotransformation product is the novel metabolite deoxynivalenol-8,15-hemiketal-7-O-β-_D_-glucoside (HKDON7G) (Figure 6).

**Table 1:** ^1^H (400 MHz; *J* in Hz) and ^13^C (100 MHz) NMR Data for Deoxynivalenol-8,15-hemiketal-7-*O*-β-d-glucoside (HKDON7G) and deoxynivalenol-7-*O*-β-d-glucoside (DON7G) in CD_3_OD^a^

	DON-8,15-hemiketal-7-O-β-d-glucoside (HKDON7G)		DON-7-O-β-d-glucoside (DON7G)	
**position**	**δC, type**	**δH (J in Hz)**	**δC, type**	**δH (J in Hz)**
2	82.6, CH	3.43, d (4.6)	82.1, CH	3.49, d (4.6)
3	70.0, CH	4.38, dt (11.1, 4.6)	69.7, CH	4.38, dt (11.1, 4.6)
4	45.2, CH_2_	1.94, dd (14.4, 11.1)	45.1, CH_2_	2.32, dd (14.5, 4.2)
		1.70, dd (14.4, 4.5)		1.94, dd (14.4, 11.1)
5	45.0, C		47.2, C	
6	54.9, C		54.1, C	
7	83.6, CH	4.16, s	78.7 CH	5.12, s
8	106.5, C		201.3, C	
9	143.2, C		137.6, C	
10	122.9, CH	5.44, dd (4.1, 1.8)	139.0, CH	6.56, dd (5.7, 1.6)
11	77.6, CH	4.78, dd (4.1, 1.8)	72.5, CH	4.95, d (5.5)
12	67.5, C		66.9, C	
13	48.7, CH_2_ o	3.83, d (3.4)	49.0, CH_2_ o	3.78, d (3.6)
		2.95, d (3.4)		3.00, d (3.6)
14	15.3, CH_3_	1.11, s	15.0, CH_3_	1.08, s
15	67.5, CH_2_	4.16, d (8.5)	62.3, CH_2_	3.95, d (11.0)
		3.37, d (8.5)		3.74, m
16	16.2, CH_3_	1.77, bs	15.5, CH_3_	1.80, bs
1′	104.8, CH	4.73, d (8.0)	103.7, CH	4.96, d (7.9)
2′	75.6, CH	3.25, m	76.1, CH	3.24, m
3′	78.4, CH	3.40, m	78.5, CH	3.40, m
4′	71.5, CH	3.28, m	71.7, CH	3.26, m
5′	78.5, CH	3.30, m	78.5, CH	3.30, m
6′	62.7, CH_2_	3.92, m	62.8, CH_2_	3.92, m
		3.72, m		3.72, m

a o, overlapping signal; HKDON7G by NMR (*H* = 1, 1.0 integration); (2) DON7G by NMR (*H* = 1, 0.67 integration).

Evaluation of the NMR data (Table 1 and Figure 6) for the minor structural isomer showed resonances consistent with DON as its conventional enone tautomer and a single glucopyranosyl moiety (Table 1). Accordingly, a single carbonyl is observed at δ 201.3 (C-8), an olefinic signal at δ 6.56 (dd, 5.7, 1.6; H-10), and HMBC correlations were observed from δ 5.12 (s; H-7) and δ 1.80 (bs; H-16) to the C-8 carbonyl. A larger coupling constant consistent with the DON enone tautomer was observed for one of the H-15 diastereotopic methylene signals at δ 3.95 (d, 11.0). Additional HMBC correlations were observed from H-7 to C-8, C-6 (δ 54.1) and C-15 (δ 62.3). The anomeric proton coupling constants at δ 4.96 (d, 7.9: H-1′) and corresponding carbon signal at 103.7 (C-1′) established the β*-*configuration of the glycosidic linkage akin to the major isomer, HKDON7G. An HMBC correlation was observed from H-1′ to C-7 connecting the glucopyranosyl moiety to the trichothecene scaffold. These NMR data, together with the MS/MS spectra, establish the structure of the minor isomer for the YjiC biotransformation product in CD_3_OD as deoxynivalenol-7-O-β-_D_-glucoside (DON7G).

Previous studies have shown that DON exists in equilibrium with a hemiketal between the C-8 and C-15 positions. This equilibrium occurs in solution and is heavily skewed toward free DON (enone form).^37,38^ A quantitative NMR study revealed that the hemiketal isomer of DON constitutes 11.1% of the total DON in D_2_O.^36^ This equilibrium distribution indicates that the DON-8,15-hemiketal has a standard Gibbs free energy (ΔG°) approximately +5.2 kJ/mol higher than the classic enone structure under the reported conditions. Interestingly, the DON hydroxyl moiety at position C-7 unlike positions C-3 and C-15, has been reported to be unreactive, with both enzymatic and synthetic approaches failing to demonstrate modifications at this site.^39−41^ The apparent inertness of the C-7 hydroxyl group and the low abundance of the DON-8,15-hemiketal form in solution likely share a common cause: the C-7 hydroxyl group engages in a strong intramolecular hydrogen bond, acting as a donor to the C-8 carbonyl oxygen. DFT calculations by Nagy et al. revealed that the low-energy conformations of DON all feature an intramolecular hydrogen bond between the C-8 carbonyl oxygen (acceptor) and the C-7 hydroxyl group (donor).^42^ Subsequent NMR studies conducted by Foroud et al. provided experimental evidence supporting this computational observation.^43^ They measured a significantly slower hydrogen exchange rate of 0.04 s^–1^ for the C-7 hydroxyl proton compared to the exchange rates of 0.12 s^–1^ for both the C-3 and C-15 hydroxyl protons. They were able to experimentally demonstrate the presence of a strong hydrogen bond between the C-7 hydroxyl and the carbonyl oxygen at position C-8. To form the 8,15-hemiketal, disruption of this strong intramolecular hydrogen bond is required. This hydrogen bonding interaction not only stabilizes the predominant enone form of DON but also significantly reduces the nucleophilicity of the hydroxyl C-7 oxygen making it less reactive toward electrophiles, explaining why it has previously been considered unreactive. Upon glycosylation of the hydroxyl at C-7 by YjiC, the resulting oxygen atom loses its capacity to serve as a hydrogen bond donor to the carbonyl moiety at C-8. This critical alteration in the intramolecular hydrogen bonding network impacts the relative energies of the molecule’s tautomeric forms. Consequently, the 8,15-hemiketal tautomer becomes energetically more favorable as compared to the DON enone isomer and shifts the equilibrium toward its formation. This shift is further highlighted by the relatively minor amount of the diagnostic product ion at *m*/*z* 427.1610 observed in the MS/MS spectrum of the YjiC-catalyzed biotransformation product (Figure 5A). This product ion could only occur when the YjiC-catalyzed product exists in its energetically less favorable DON7G enone tautomer in solution. The ability of YjiC to modify the hydroxyl at C-7 of DON is novel and likely involves the disruption of the strong intramolecular hydrogen bond network. Further biochemical and structural characterization is necessary to better understand how YjiC interacts with DON to glycosylate the C-7 hydroxyl position.

### HKDON7G Is Not Toxic to Duckweed

**Figure 7:**
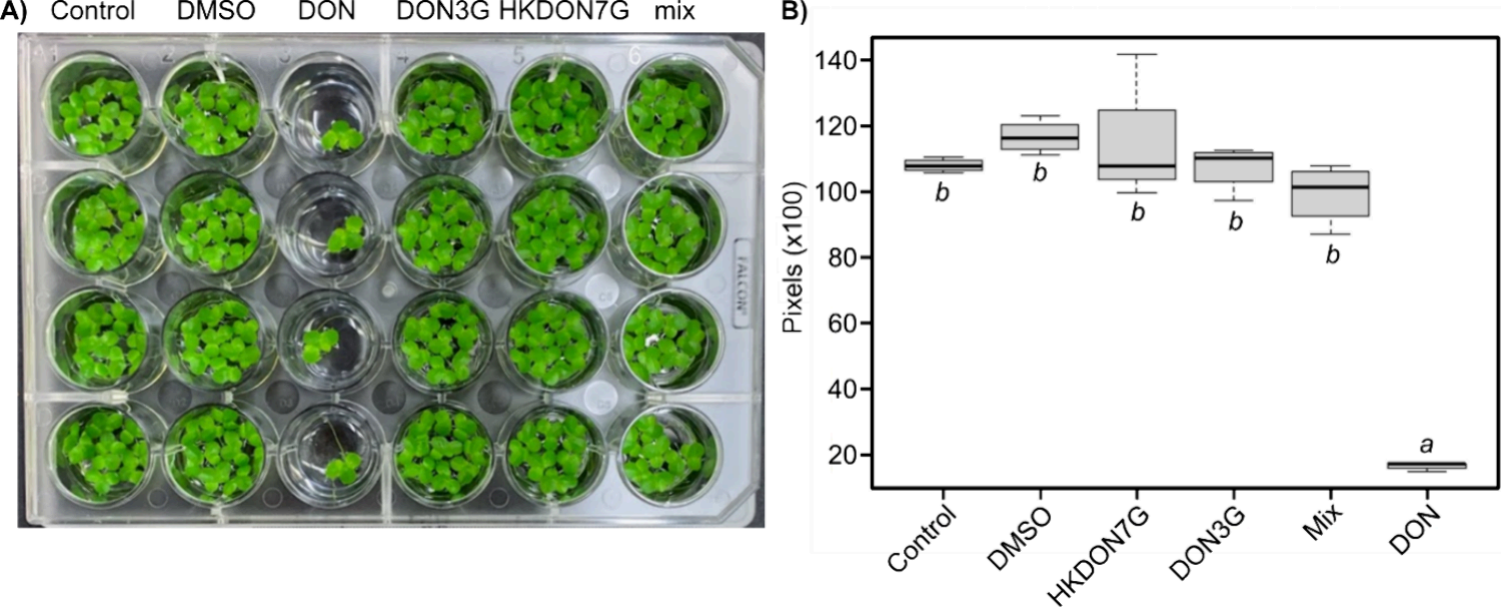
HKDON7G is nontoxic to duckweed. (A) Duckweed (*Lemna minor*) grown in the presence of 20 μM DON, DON3G, HKDON7G, and an unpurified YjiC-catalyzed reaction mixture (mix). Control = no DMSO and no toxin, DMSO = no toxin control. (B) Quantification of growth from panel (A). Number of pixels measured. Error bars represent standard deviation (*n* = 4). Conditions bearing the same italicized letters are not statistically different from one another as determined by the Tukey method for one-factor ANOVA (*p* < 0.05).

To investigate the phytotoxicity of HKDON7G, duckweed (*Lemna minor*) was grown in the presence of 20 μM DON, DON3G, HKDON7G, and an unpurified YjiC-catalyzed reaction containing HKDON7G and minor amounts of the byproducts DON3G and diglycosylated HKDON3,7G (Figure 7). We monitored plant growth for each treatment by measuring total frond surface area as previously reported.^44^ We employed duckweed since it is a model organism^45^ and because DON is phytotoxic. We observed a significant reduction in the growth of the plant in the presence of DON as compared to the negative controls (Figure 7). Conversely, we observed no significant growth reduction in the presence of DON3G, pure HKDON7G, or the YjiC-catalyzed mixture. These data indicate that like DON3G,^10^ HKDON7G is not phytotoxic.

### HKDON7G Is More Stable than DON3G

**Figure 8:**
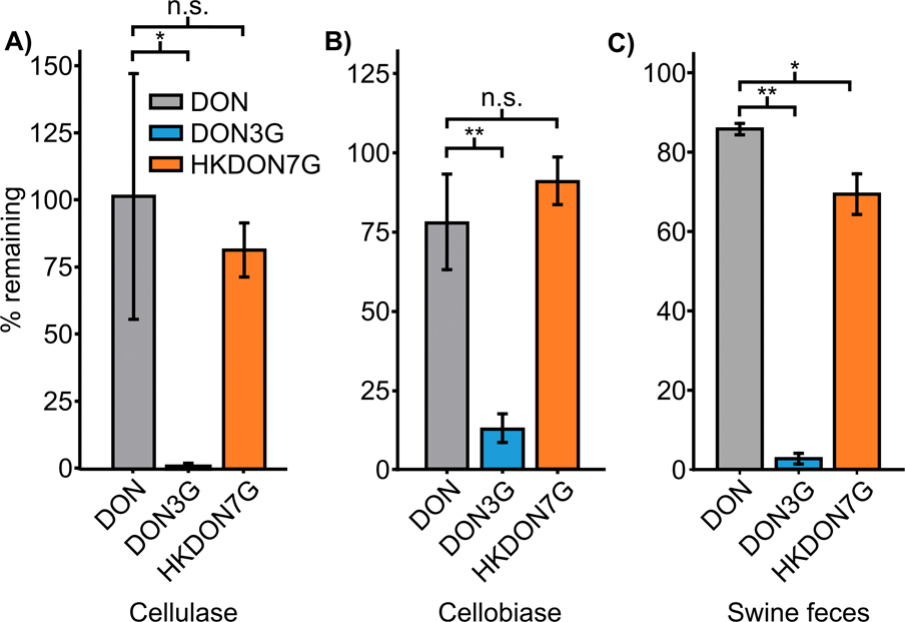
Percentage remaining (as determined via LC-MS extracted ion peak areas) of initial levels of DON (gray), DON3G (blue), and HKDON7G (orange) when incubated in the presence of either (A) cellulase (10 U/mL), (B) Cellobiase (1.3 U/mL), or (C) a swine fecal slurry. Error bars represent standard deviation (*n* = 3). * = *p* < 0.05, ** = *p* < 0.01 as determined via *t* test. n.s. = not significantly different.

While the glycosylation of DON is a known detoxification mechanism in plants, several studies have shown that DON3G is readily hydrolyzed back to DON by various enzymes and bacterial cultures in animal guts.^46^ In order to test the stability of HKDON7G, we incubated the purified product in the presence of two previously identified enzyme preparations capable of hydrolyzing DON3G into DON: cellulase (Figure 8A) and cellobiase (Figure 8B).^46^ We observed no significant hydrolysis of HKDON7G in the presence of either enzyme, while 98% and 86% of DON3G was hydrolyzed by cellulase (10 U/mL) and cellobiase (1.3 U/mL), respectively. To further test the stability of HKDON7G, we incubated it anaerobically for 24 h in a slurry of freshly collected pig feces and compared its hydrolysis to DON3G. Following incubation, we observed a 95% reduction in total DON3G levels (Figure 8C), and only a 25% reduction in HKDON7G levels. These data indicate that HKDON7G is significantly more stable and resistant to hydrolysis than DON3G under these conditions. We hypothesize the increased difficulty in hydrolysis of HKDON7G compared to DON3G arises from the formation of the stabilizing hemiketal, which would sterically hinder enzymatic access to the anomeric carbon prior to displacement of the aglycone moiety.

Here we reported the isolation of a strain of *B. subtilis* that is capable of biotransforming DON into the novel metabolite HKDON7G. This bacterium was isolated from the soil of maize fields in Ontario suffering from a major epidemic of Gibberella ear rot. Proteomic analysis of the glycosylation-activity-enriched fractions from *B. subtilis*_AAFC1 revealed that YjiC was the enzyme responsible for the observed DON biotransformation. NMR and LC-MS/MS data indicate that purified recombinant YjiC employs UDP-glucose to glycosylate the 7 position of DON. C-7 glycosylation drives hemiketal formation between positions C-8 and C-15, leading to the formation of HKDON7G, a novel DON biotransformation product. HKDON7G exists in equilibrium with the nonhemiketal enone form (DON7G), however, the equilibrium is strongly biased toward hemiketal formation. HKDON7G was shown to lack phytotoxicity toward duckweed and to be significantly more resistant to hydrolytic degradation compared to DON3G. This study demonstrates that YjiC shows promise as a tool for the detoxification of DON contaminated food and feed and warrants further investment and testing.

The *Bacillus* genus, notably *B. subtilis*, displays a remarkable metabolic versatility and stress tolerance that likely arose from inhabiting diverse environments, including soils and digestive tracts.^47^ It is not surprising certain species within the *Bacillus* genus have evolved the ability to detoxify DON.^27,28^ Nevertheless, this is the first report of *B. subtilis* generating the novel DON biotransformation product HKDON7G. The product is generated via a unique mechanism that targets the hydroxyl moiety at position C7 of the trichothecene backbone, a position previously hypothesized to be unreactive.^39−41^ Despite the ubiquity of *B. subtilis* and the well characterized role of YjiC as a glycosyltransferase involved in xenobiotic detoxification,^32,48^ their identification herein is a testament to the robustness of our LC/MS-based discovery pipeline to identify novel mycotoxin biotransformation activities from previously well-characterized microbes and enzymes.^29,30^

## Conclusions

Our results demonstrate that the *B. subtilis* glycosyltransferase YjiC glycosylates DON at the 7-hydroxyl position, generating the novel metabolite HKDON7G. This novel metabolite is nontoxic to duckweed and is significantly more resistant to hydrolysis compared to DON3G, a known plant detoxification product of DON. Future studies will focus on understanding the specificity of YjiC toward DON and other trichothecenes, including the emerging NX-toxin.^49^ Structure activity studies with different trichothecenes will aid in elucidating YjiC’s ability to target the C-7 hydroxyl position. It is likely solvent affects the equilibrium of hemiketal formation following glycosylation at C-7 by YjiC. Further NMR structural studies in additional solvents, including D_2_O and DMSO, are necessary. HKDON7G’s lack of toxicity toward duckweed is promising and supports the need for further toxicity research using appropriate cell lines and animal models. Lastly, experiments to determine the optimal conditions for deploying YjiC for the remediation of contaminated food and feed are needed to support its commercial development.

## Materials and Methods

### Application of the LC-HRMS-Based Discovery Pipeline for DON Biotransformation Activity

For this study, agricultural soil samples were collected from fields in Southern Ontario, Canada using a soil probe, placed in sealed plastic bags, and stored at −20 °C prior to testing. Nutrient Broth (NB), Luria–Bertani (LB) broth, Malt Extract Broth (MEB), and Yeast Extract Sucrose (YES) (yeast extract supplemented with sucrose (150 g/L) and MgSO_4_.7H_2_O (0.5 g/L) (BD Biosciences, Mississauga, ON, Canada) broth were used for microbial enrichment. Full strength and 1/100 dilutions of the media were used as culture media. The 1/100 dilutions were prepared with inorganic salt culture media as previously described.^50^ Soil samples (0.5 g) were incubated with 2.5 mL of culture media containing 300 μM DON (Triple Bond, Guelph, ON, Canada) for 1 week. All cultures were incubated at 28 °C and shaken aerobically. Subsequently, 100 μL of the incubated cultures were transferred into fresh media containing 300 μM DON each week for 4 weeks total. Following this, 100 μL of each sample was quenched with 900 μL methanol (MeOH), filtered using a 0.45 μm PTFE syringe filter (Fisher Scientific, Lawn, NJ, USA), and DON biotransformation assessed using liquid chromatography - high resolution mass spectrometry (LC-HRMS) analysis (see section below). Mixed microbial cultures that displayed DON biotransformation capabilities were serially diluted and plated onto their respective media agar plates. Single colonies were inoculated into various liquid media (either full strength or 1/100 dilutions), incubated for 1 week at 28 °C with shaking, and retested to confirm their ability for DON degradation.

### LC-HRMS Analysis for Identification of DON Biotransformation Activity

Microbial and recombinant enzymatic activity were monitored using LC-HRMS on a Q-Exactive Orbitrap mass spectrometer (Thermo Fisher Scientific, Waltham, MA, USA) with a heated electrospray ionization (HESI) source coupled to an Agilent 1290 ultrahigh-performance liquid chromatography system. The samples were analyzed in negative ionization mode with DON being monitored as the formate adduct [DON + HCOO]^−^ at *m*/*z* 341.1242. Glycosylated DON metabolites were screened as either the [M - H]^−^ or [M + HCOO]^−^ adducts at *m*/*z* 457.1715 and 503.1770 respectively. Chromatographic separation was performed using an Eclipse Plus RRHD C-18 column (2.1 × 50 mm, 1.8 μm; Agilent, Mississauga, ON, Canada) maintained at 35 °C with 0.1% formic acid (mobile phase A), and acetonitrile with 0.1% formic acid (mobile phase B; Optima grade, Fisher Scientific, Lawn, NJ, USA) at a flow rate of 0.3 mL/min. The gradient was held at 0% B for 30 s, increased to 100% B over 3 min, held at 100% B for 2.5 min, decreased to 0% B over 30 s and finally held at 0% B for one min. For each sample, 5 μL was injected. HESI conditions were as follows: capillary temperature, 400 °C; sheath gas, 17 units; auxiliary gas, 8 units; probe heater temperature, 400 °C; S-Lens RF level, 45; capillary voltage 3.5 kV. All samples were analyzed using a top five, data-dependent acquisition method. The full scan was acquired between a mass range of *m*/*z* 175–900 at 35,000 resolution with an automatic gain control (AGC) of 3 × 10^6^ and a maximum injection time (IT) of 128 ms. The MS/MS scans were acquired at 17,500 resolution, AGC of 1 × 10^5^, maximum IT of 50 ms, intensity threshold of 4 × 10^3^, NCE of 22 and an isolation window of 1.2 *m*/*z*. All data was analyzed using Thermo Xcalibur QualBrowser software (version no. 4.3).

### Bacterial Identification and Sequencing

Candidate DON biotransforming bacterial strains were initially identified via Sanger sequencing of the 16S rRNA PCR products prior to full genomic sequencing. For PCR, bacterial genomic DNA was extracted using the DNeasy UltraClean Microbial Kit (QIAGEN, Toronto, Canada). PCR amplification of the 16S rRNA gene was performed using the primers 27F (5′-AGAGTTTGATCMTGGCTCAG-3′) and 1492R (5′-GGTTACCTTGTTACGACTT-3′). The PCR reaction occurred as follows: 95 °C for 3 min; 30 cycles of 95 °C for 1 min, 55 °C for 30 s, 72 °C for 1 min; and finally 72 °C for 5 min. The PCR product was purified using a Quick PCR Purification Kit (PureLink, Millipore Sigma, Mississauga, ON, Canada) and sequenced at the London Regional Genomics Centre (Western University, London, ON, Canada). 16S rRNA sequences were BLAST searched against the NCBI 16S rRNA sequences (Bacteria and Archaea) database. Initial hits indicated the bacterium belonged to the *Bacillus* genus. To sequence the full genome of this bacterium, an individual colony was cultured overnight with shaking at 28 °C in 5 mL of NB media. Cells were pelleted and sent on ice to CD Genomics (https://www.cd-genomics.com/, Shirley, NY, USA) for whole genome sequencing and assembly. The total genome length was 4,081,710 bp, consisting of one long (4,060,208 bp) and one short (21,502 bp) contig. The GC content of the genome was 43.9%. The genome was converted into a proteome.FASTA format for subsequent proteomics analysis.

### Biochemical Enrichment of DON Biotransformation Activity from *B. subtilis*_AAFC1

A single colony of *B. subtilis*_AAFC1, (IDAC accession no. 310724–01, identified as described above), was inoculated into 25 mL of NB media and incubated aerobically at 37 °C for 24 h with shaking. Afterward, the culture was inoculated into 1 L of NB media and grown aerobically at 37 °C for 24 h with shaking. The culture was centrifuged and the pellet resuspended in 50 mM HEPES (pH 7.0), 150 mM NaCl, 1.43 mM β-mercaptoethanol, 2 mM EDTA, and 0.2 mg/mL lysozyme. The resuspended pellet was lysed by sonication for a total of 2.5 min in 30 s on/off bursts, and the lysate supernatant was clarified via centrifugation. The lysate supernatant was then subjected to a stepwise 0–30%, 30%–60%, and a 60%–90% (w:v) (NH_4_)_2_SO_4_ precipitation. Precipitated pellets were resuspended in 50 mM HEPES (pH 7.0) and dialyzed against 50 mM HEPES (pH 7.0) at 4 °C overnight. The most active fraction (as determined via LC-HRMS analysis) was loaded onto a 1 mL HiTrap Q HP Column (GE Healthcare, Mississauga, ON, Canada) equilibrated in 50 mM HEPES (pH 7.0), and 50 mM NaCl (Buffer A). Protein was eluted using a linear 50–1000 mM NaCl gradient in Buffer A over 10 column volumes. Fractions displaying DON biotransformation activity were pooled and loaded onto an Enrich SEC 650 column (BIO-RAD, Mississauga, ON, Canada) equilibrated in 50 mM HEPES and 150 mM NaCl (pH 7.0). Fractions displaying activity were dialyzed against 50 mM ammonium bicarbonate (pH 8.0) prior to proteomics analysis. To test for DON biotransformation activity during biochemical enrichment, all fractions were administered 1 mM DON and 10 mM UDP-glucose and allowed to incubate overnight at room temperature prior to LC-HRMS analysis.

### Proteomics Sample Preparation and Analysis

Following biochemical enrichment of DON biotransformation activity, 200 μL aliquots of purified protein fractions were dissolved in 5 mM DTT and incubated at 60 °C for 30 min. At room temperature, the reduced proteins were alkylated in darkness using iodoacetamide at a concentration of 10 mM for 15 min, after which an additional 10 mM DTT was then added. The proteins were digested by incubating with 200 ng of Trypsin (Thermo Scientific Pierce sequencing grade, Rockford, IL, USA) overnight at 34 °C. The digestion was quenched by adding formic acid to a final concentration of 0.1% (v:v). The tryptic peptides were then passed through a 1 mL, 30 mg sorbent, Waters Oasis HLB SPE cartridge (Milford, MA, USA), which was activated with methanol and preconditioned with LC/MS grade H_2_O containing 0.1% (v:v) formic acid. The cartridges were dried under vacuum for 5 min and the peptides were then eluted into fresh 1.5 mL microcentrifuge tubes by addition of 400 μL of 70% (v:v) acetonitrile. The samples were subsequently dried by vacuum centrifugation. The samples were then reconstituted in 200 μL of water/acetonitrile/formic acid (95/4.9/1) and transferred to 250 μL polypropylene HPLC vials.

The purified peptide digests were separated using an Easy-nLC 1000 nanoflow HPLC system fitted with a 2 cm Acclaim C18 PepMap trap column and a 75 μm x 25 cm Acclaim C18 PepMap analytical column (Thermo Scientific, USA). The flow rate was held at 300 nL/min throughout the run and 10 μL of the digest was injected. The mobile phase A (LC/MS Optima water, 0.1% formic acid) began at 97% and was decreased to 90% over 4 min. Peptides were then eluted with a linear gradient of 10% to 40% mobile phase B (LC/MS Optima acetonitrile, 0.1% (v:v) formic acid) over 150 min, followed by 40%–90% over 10 min, and maintained constant for an additional 10 min. Each sample was then analyzed using a top ten, data-dependent acquisition method using a Thermo Q-Exactive Orbitrap mass spectrometer. The nanospray voltage was set at +2.8 kV, capillary temperature at 275 °C, and the S-lens radio frequency (RF) level at 70. The full scan was acquired between a mass range of *m*/*z* 340–1800 at 70,000 resolution with an automatic gain control (AGC) of 1 × 10^6^ and a maximum injection time of 256 ms. The MS/MS scans were acquired at 17,500 resolution, AGC of 1 × 10^6^, maximum IT of 110 ms, intensity threshold of 2 × 10^5^, normalized collision energy of 30 and an isolation window of 2 *m*/*z*. Unassigned, singly charged, and peptides with >5 charges were not selected for MS/MS, and a 15 s dynamic exclusion was used. Thermo.raw files were converted to.mgf with Proteowizard (v2) and MS/MS scans were searched against the target proteome (as determined via genomic sequencing) using X! Tandem search algorithm operated from the SearchGUI v.3.3.3 interface and processed in PeptideShaker v1.16.26. A 3 ppm precursor ion mass error and a 0.02 Da product ion error were used along with oxidation of methionine as a variable modification. A 1% false discovery (FDR) rate was used at the protein, peptide, and peptide spectrum match level.

### Recombinant Protein Production and Purification

The sequenced gene from *B. subtilis*_AAFC1 was synthesized and codon optimized for expression in *E. coli* by Twist Biosciences (San Francisco, CA, USA). The gene was placed into the pET His6-MBP TEV LIC cloning vector (Addgene plasmid #29653, a gift from Scott Gradia) to produce an N-terminally 6x His-tagged MBP-fusion protein. The recombinant gene was overexpressed in *E. coli* BL21(DE3), and protein production was induced at 16 °C overnight with shaking via the addition of 0.5 mM IPTG once the OD_600_ of the culture reached 1.0. Following expression, the cell pellet was resuspended in 50 mM Tris-HCl (pH 8.0), 150 mM NaCl, and 5 mM imidazole. Cells were lysed via sonication as described above, and the recombinant gene was enriched from clarified cell lysate via Ni-NTA affinity. Protein was eluted from the column in buffer containing 50 mM Tris-HCl (pH 8.0), 150 mM NaCl, and 400 mM imidazole. The MBP fusion tag was cleaved via TEV protease digestion at a 1:50 ratio of TEV:protein overnight at 4 °C. The reaction was then subjected to an Ni-NTA column again, and the column flow-thru was collected and subjected to gel permeation chromatography using a HiLoad 16/600 Superdex 200 prep grade column (Cytiva, Mississauga, ON, Canada) equilibrated in 50 mM Tris-HCl (pH 8.0) and 150 mM NaCl. Fractions containing active enzyme were again pooled and protein concentration was determined via absorbance at 280 nm using the protein’s extinction coefficient as determined via Expasy ProtParam.^51^ Enzyme was flash frozen and stored at −80 °C until further use.

### Isolation of Enzyme-Catalyzed DON Glycosylation Products

To test for DON biotransformation activity, 1 μM purified recombinant enzyme was incubated with 1 mM DON, 5 mM UDP-glucose, and 10 mM MgCl_2_ in buffer containing 50 mM Tris-HCl (pH 8.0) and 150 mM NaCl. Reactions were performed at 37 °C and allowed to proceed for 16 h, prior to being quenched with ice-cold MeOH and analyzed via LC-HRMS. To scale up production of the enzyme-catalyzed product, 20 mL reactions consisting of 3.375 μM purified recombinant enzyme, 1 mM UDP-glucose, 3.375 mM DON, 10 mM MgCl_2_, in 50 mM Tris-HCl (pH 8.0) and 150 mM NaCl were incubated for 24 h at 37 °C. The resulting mixture was purified using reversed-phase flash column chromatography on an automated Büchi system with a 12 g C18 Sepacore column (Büchi, Flawil, Switzerand). The gradient using 0.1% (v:v) formic acid in water (A) and 0.1% formic acid in acetonitrile (B) started at 1% B for 2 min before increasing to 25% B over 10 min. The gradient increased to 100% over 3 min and was held for 3 min before returning to 2%. The enzymatic product was monitored at 218 and 254 nm and fractions were collected every 10 mL. Fractions were screened by LC-HRMS and those containing the desired product were combined and evaporated to dryness first using a N_2_ evaporator to remove organic solvent, followed by freeze-drying.

Homonuclear (^1^H, ^13^C, COSY) and heteronuclear (HSQC and HMBC) NMR spectra of the purified enzyme-catalyzed DON glycosylation product (5.0 mg) were recorded with a 400 MHz JEOL ECZS Spectrometer (Peabody, MA) with an autotuning probe. The purified product was dissolved in CD_3_OD (CDN Isotopes, Pointe-Claire, Quebec) and referenced to the appropriate solvent peak (δ_H_ 3.31 and δ_C_ 49.0). NMR spectra are reported in the Supporting Information.

### Structural Characterization of Enzyme-Catalyzed DON Glycosylation Products

The main enzyme-catalyzed DON biotransformation product, along with commercially obtained DON3G (Romer Laboratories; Vancouver, BC, Canada) and synthetically produced DON15G (Figure 1) were analyzed using targeted high-resolution LC-MS/MS on the Q-Exactive Orbitrap platform with conditions as described above with some modifications. The different reaction products were resolved chromatographically using an Eclipse Plus RRHD C-18 column (2.1 × 150 mm, 1.8 μm; Agilent, Mississauga, ON, Canada) maintained at 35 °C. Mobile phase A was held at 100% for 45 s after which mobile phase B was increased to 15% over 30 s. Mobile phase B was then gradually increased from 15% to 32% over 5.75 min to obtain optimal resolution of the glycosylation products. Mobile phase B was increased to 100% over 1 min and held for 2.5 min before returning to 0% over 30 s. MS/MS was performed in negative ionization mode on both the [M-H]^−^ or [M+HCOO]^−^ adducts at *m*/*z* 457.1715 and 503.1770 respectively at a NCE of 25, resolution of 17,500, AGC of 3 × 10^6^, maximum IT of 64 ms and an isolation window of 1.2 *m*/*z*.

DON15G was prepared synthetically via a Koenigs-Knorr reaction as previously described^52^ for the production of DON-glucuronides with DON and acetobromo-α-d-glucuronic acid methyl ester being substituted for 3ADON and acetobromo-α-d-glucose, (Millipore Sigma, St Louis, MO) respectively. After 144 h, 1 mL of the reaction supernatant was transferred to an 8 mL glass scintillation vial, dried under an N_2_ evaporator and reconstituted in 1 mL of 80% (v:v) acetonitrile. The product was deacetylated by adding 5 μL of 6 M KOH and incubating for 1 h. Ten μL of the basic solution containing the deacetylated product was transferred to a 2 mL amber glass HPLC vial containing 990 μL of 20% (v:v) acetonitrile and analyzed by high resolution LC-MS/MS.

### Lemna Minor Toxicity Assay

*Lemma minor* strain CPCC 490 (Phycological Culture Collection, Kitchener, ON, Canada) was maintained in a growth cabinet using Hoagland’s E+ medium according to.^44^ Assay conditions were carried out in 24-well TC-treated Costar plates (Millipore Sigma, St. Louis, MO, USA) using 1.25 mL of Hoagland’s E+ medium without sucrose or tartaric acid. Stock concentrations of DON, DON3G, HKDON7G, and the unpurified enzyme product mixture were prepared in Optima acetonitrile (Fisher Scientific, Mississauga, ON, Canada). Test conditions included quadruplicate biological replicates of controls (Hoagland’s E+ assay media only), DMSO controls (assay media with 0.1% (v:v) DMSO), 20 μM DON, 20 μM DON3G, 20 μM HKDON7G, and 20 μM enzyme product mixture. 0.1% (v:v) DMSO was added to each well as a solvent reservoir prior to the addition of the stock concentrations of DON, DON3G, HKDON7G, and the enzyme product mixture. The acetonitrile in each well was dried before the addition of Hoagland’s assay media up to 1.25 mL. Duckweed plants with three fronds were selected and added to each well. The 24-well assay plate was put into a 3D-printed plastic sleeve and incubated in a growth cabinet under consistent temperature and lighting conditions for 7 days.^44^ After 7 days, the assay plate was imaged and subjected to the Python pixel analysis pipeline (available at https://github.com/sumarah-lab/Duck-Weed-Image-Analysis) as adapted by.^44^ Following image analysis, duckweed fronds were removed from each well, freeze-dried and weighed for biomass. Single-factor ANOVA with Tukey’s posthoc testing (*p* < 0.05) was used to evaluate significance from the green pixel count and dry biomass from each condition using R. Data were visualized using box and whisker plots prepared in R (v. 4.4.1).

### Stability and Hydrolysis Assessment

HKDON7G and DON3G were tested for enzymatic hydrolysis with cellulase (≥700 U/g, *Trichoderma reesei,* Millipore Sigma, St. Louis, MO, USA) and cellobiase (1300 U/g, *Aspergillus niger*, Millipore Sigma, St. Louis, MO, USA) according to the approach by.^46^ Reaction mixtures in 100 μL volumes containing either 1 μM HKDON7G or 1 μM DON3G were incubated with either 10 U/mL cellulase (14.3 mg/mL; 0.1 M sodium acetate, pH 5) or 1.3 U/mL cellobiase (1 mg/mL; 50 mM sodium phosphate, pH 6, 5 mM EDTA) for 24 h at 37 °C with gentle shaking. The reactions were quenched by the addition of 100 μL MeOH, vortexed for 15 s and centrifuged. From this, 190 μL of the reaction supernatants were transferred to 250 μL polypropylene HPLC vials for analysis by LC-HRMS.

### Pig Fecal Sample Stability Assay

Adapted methods for the DON metabolite hydrolysis stability assays are described in.^23^ Briefly, feces from several pigs were collected directly after defecation at the University of Guelph Animal Research Center. Feces were transported to the AAFC London Research and Development Centre and stored anaerobically at −80 °C in 10 mL of 70% PBS (pH 7.0), 30% (v:v) glycerol until further use. Samples were thawed and spun down at 4,000*g* for 15 min. The supernatant was discarded, and the pellet was suspended with 1 mL of anaerobic M2 medium.^24^ Tubes were incubated at 37 °C and 200 rpm for 1 h. Post incubation, 100 μL were collected and 2 mM of either HKDON7G, DON3G, or DON were added. Samples were incubated at 37 °C for 24 h and analyzed by LC-HRMS for stability.

## Supporting Information Available

Table S1 (LC-MS/MS proteomics results) and various NMR spectra for HKDON7G (PDF)

## Abbreviations

3ADON: 3-acetyl-deoxynivalenol
AAFC: Agriculture & Agri-Food Canada
AGC: automatic gain control
DON15G: deoxynivalenol-15-glucoside
DON3G: deoxynivalenol-3-glucoside
DFT: density functional theory
DMSO: dimethyl sulfoxide
DON: deoxynivalenol
DON7G: DON-7-glucoside
DTT: dithiothreitol
EDTA: ethylenediaminetetraacetic acid
FDR: false discovery rate
HEPES: 4-(2-hydroxyethyl)piperazine-1-ethanesulfonic acid
HESI: heated electrospray ionization
HKDON3,7G: DON-8,15-hemiketal-3,7-diglucoside
HKDON7G: DON-8,15-hemiketal-7-glucoside
SPE: solid phase extraction
HMBC: heteronuclear multiple bond correlation
HPLC: high-performance liquid chromatography
HSQC: heteronuclear single quantum coherence
IPTG: isopropyl β-d-1-thiogalactopyranoside
IT: injection time
LB: Luria–Bertani
LC-HRMS: liquid chromatography high-resolution mass spectrometry
LIC: ligation-independent cloning
MBP: maltose binding protein
MEB: malt extract broth
MeOH: methanol
NB: nutrient broth
NCE: normalized collision energy
Ni-NTA: nickel nitrilotriacetic acid
NMR: nuclear magnetic resonance
PPM: parts per million
PTFE: polytetrafluoroethylene
RPM: revolutions per minute
RRHD: rapid resolution high definition
rRNA: ribosomal ribonucleic acid
SDS-PAGE: sodium dodecyl sulfate polyacrylamide gel electrophoresis
TEV: tobacco etch virus
Tris: tris(hydroxymethyl)aminomethane
UDP: uridine diphosphate
YES: yeast extract sucrose
