# Supplementary material for: Biotransformation of Deoxynivalenol to the Novel Metabolite Deoxynivalenol-8,15-hemiketal-7-glucoside by the Bacillus subtilis Glycosyltransferase YjiC
Source: ACS Omega. 2025 Mar 24;10(14):14496–507. doi: 10.1021/acsomega.5c01301 (PMC12004161; doi:10.1021/acsomega.5c01301)
Supplement: Supplementary file 1 — ao5c01301_si_001.pdf [file ao5c01301_si_001.pdf]

Supporting information

Biotransformation of deoxynivalenol to the novel metabolite deoxynivalenol-8,15-hemiketal-7-glucoside by the *Bacillus subtilis* glycosyltransferase YjiC

Shawn J. Hoogstra[1], Justin B. Renaud[1], David R. McMullin[2], Megan J. Kelman[1], Christopher P. Garnham[1*], and Mark W. Sumarah[1*]

[1] Agriculture and Agri-Food Canada, London Research and Development Centre, 1391 Sandford Street, London, ON, Canada, N5V 4T3

[2] Department of Chemistry, Carleton University, Ottawa, ON, K1S 5B6, Canada

*Correspondence: mark.sumarah@agr.gc.ca; Tel.: +1-226-688-6187
chris.garnham@agr.gc.ca; Tel.: +1-519-670-1732

Table S1: LC-MS/MS proteomics results showing top 30 proteins identified in Fraction iii (Figure 3C). The top ranked candidate (YjiC) is highlighted in yellow.

Main Accession	Gene Name	MW [kDa]	Possible Coverage [%]	Coverage [%]	Spectrum Counting
MGA_784	putative UDP-glucosyltransferase YjiC	43.85734295	82.14	72.7	1109.116427
MGA_891	5-methyltetrahydropteroyltriglutamate--homocysteine methyltransferase	86.74155587	85.04	63.12	247.4424402
MGA_1067	pyruvate carboxylase	127.9164718	88.33	36.41	81.16790185
MGA_2336	isocitrate dehydrogenase	46.38983705	75.89	64.78	346.9472727
MGA_2705	vegetative protein	29.01258368	100	75.48	561.2271912
MGA_2828	phosphoglycerate kinase	42.16396321	100	88.83	1177.393873
MGA_1	putative thiamine pyrophosphate-containing protein YdaP	63.01723591	76.31	66.38	470.9922077
MGA_3901	1-pyrroline-5-carboxylate dehydrogenase 2	56.38766338	89.9	68.93	256.0890421
MGA_3151	serine hydroxymethyltransferase	45.46119037	73.98	58.55	508.3018062
MGA_2658	threonine synthase	37.81350811	90.99	61.41	659.0881343
MGA_3672	glutamate--tRNA ligase	55.70155727	92.96	55.69	273.7991123
MGA_174	(R,R)-butanediol dehydrogenase	37.317527	70.52	50	1219.510309
MGA_2627	hypothetical protein	25.50803122	64.89	48.89	522.1809087
MGA_2337	citrate synthase 2	41.70249447	79.03	67.47	764.9020853
MGA_1675	aspartate aminotransferase	43.08816326	72.01	69.21	397.3347491
MGA_3173	putative fructose-bisphosphate aldolase	30.38134923	87.02	75.44	297.64437
MGA_1791	phosphopentomutase	43.97503649	75.38	63.45	388.9859877
MGA_892	major intracellular serine protease	33.82921133	87.46	57.99	229.3044338
MGA_1259	aspartate-semialdehyde dehydrogenase	37.82332897	76.88	54.91	275.1981099
MGA_480	phosphoglucomutase	64.65300202	100	52.84	73.08988568
MGA_2481	phosphoenolpyruvate carboxykinase	58.23616816	79.7	47.25	202.8036822
MGA_1830	6-phosphogluconate dehydrogenase, NADP(+)-dependent, decarboxylating	51.74249871	100	48.4	140.0453022
MGA_3649	cysteine synthase	32.80011255	89.61	51.62	302.5400947
MGA_3195	arginine--tRNA ligase	62.6426482	94.42	53.78	144.4652892
MGA_1549	putative aldehyde dehydrogenase DhaS	53.86202934	68.08	45.45	179.4490517
MGA_1437	gamma-glutamyltranspeptidase	64.14872578	72.57	28.62	72.73873892
MGA_1381	transketolase	72.25518948	83.51	34.18	156.815505
MGA_3692	elongation factor Tu	43.56584272	89.9	41.92	291.6889917
MGA_3227	phosphate acetyltransferase	34.82695782	89.78	73.37	360.8803682
MGA_2701	FeS cluster assembly protein SufB	52.69607048	84.73	47.74	233.1301079

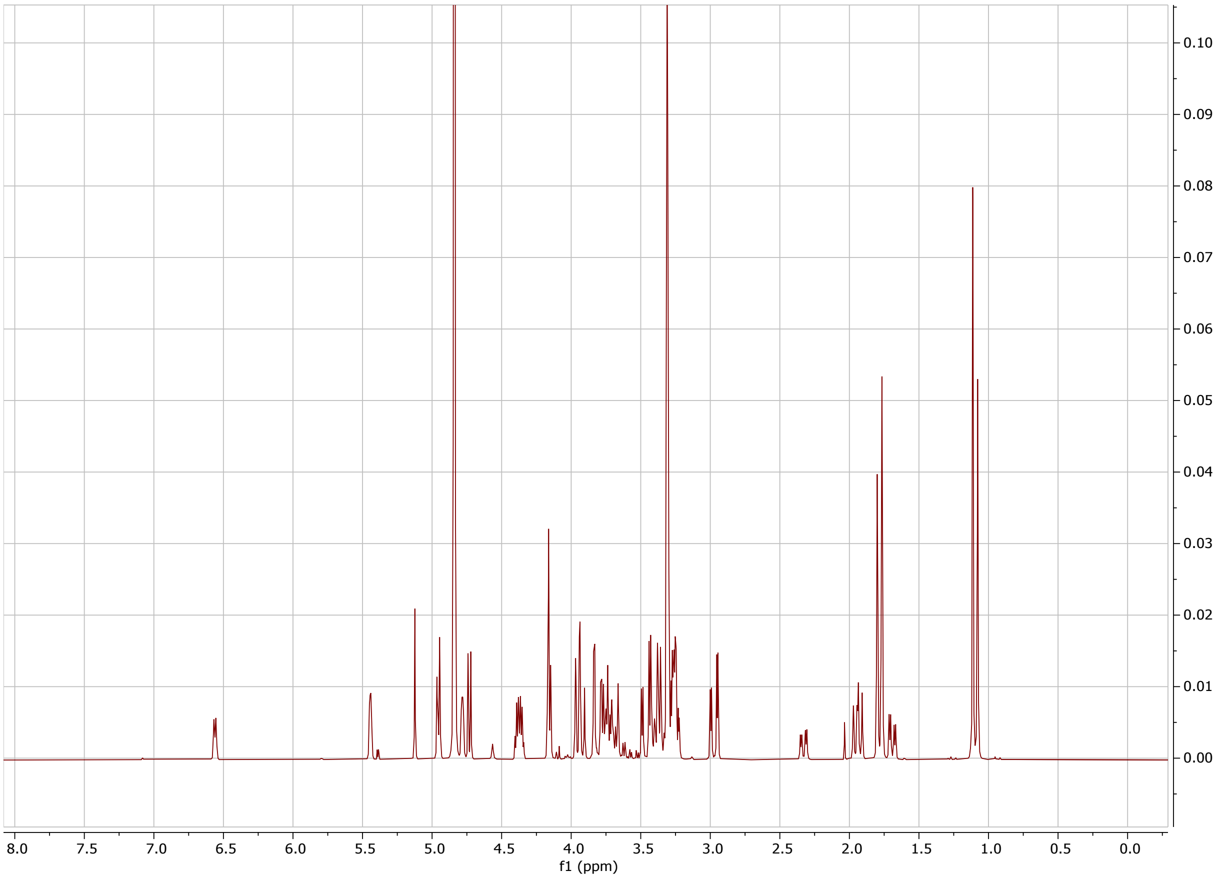

Figure S1. ^1H NMR spectrum for the YjiC biotransformation product in CD_3OD

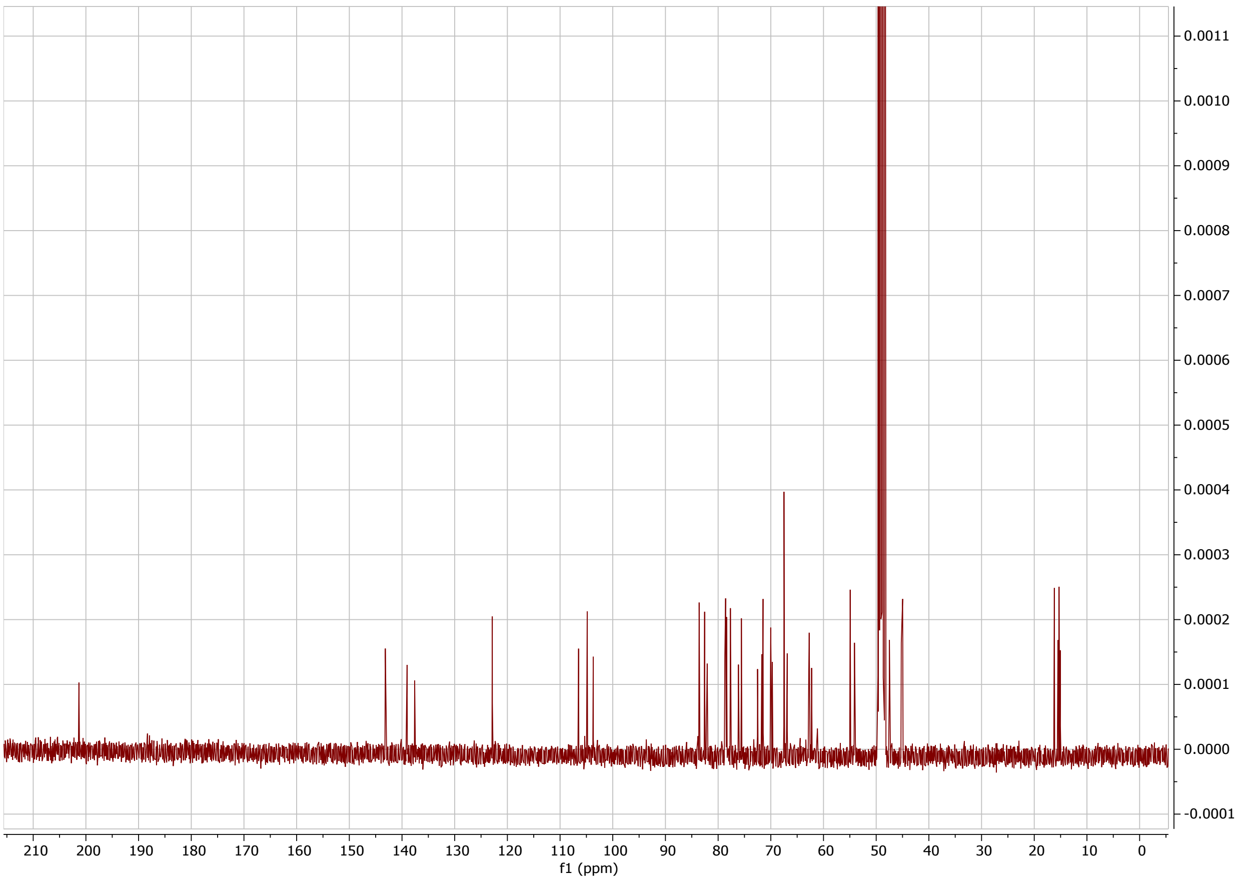

Figure S2. ^{13}C NMR spectrum for the YjiC biotransformation product in CD_3OD

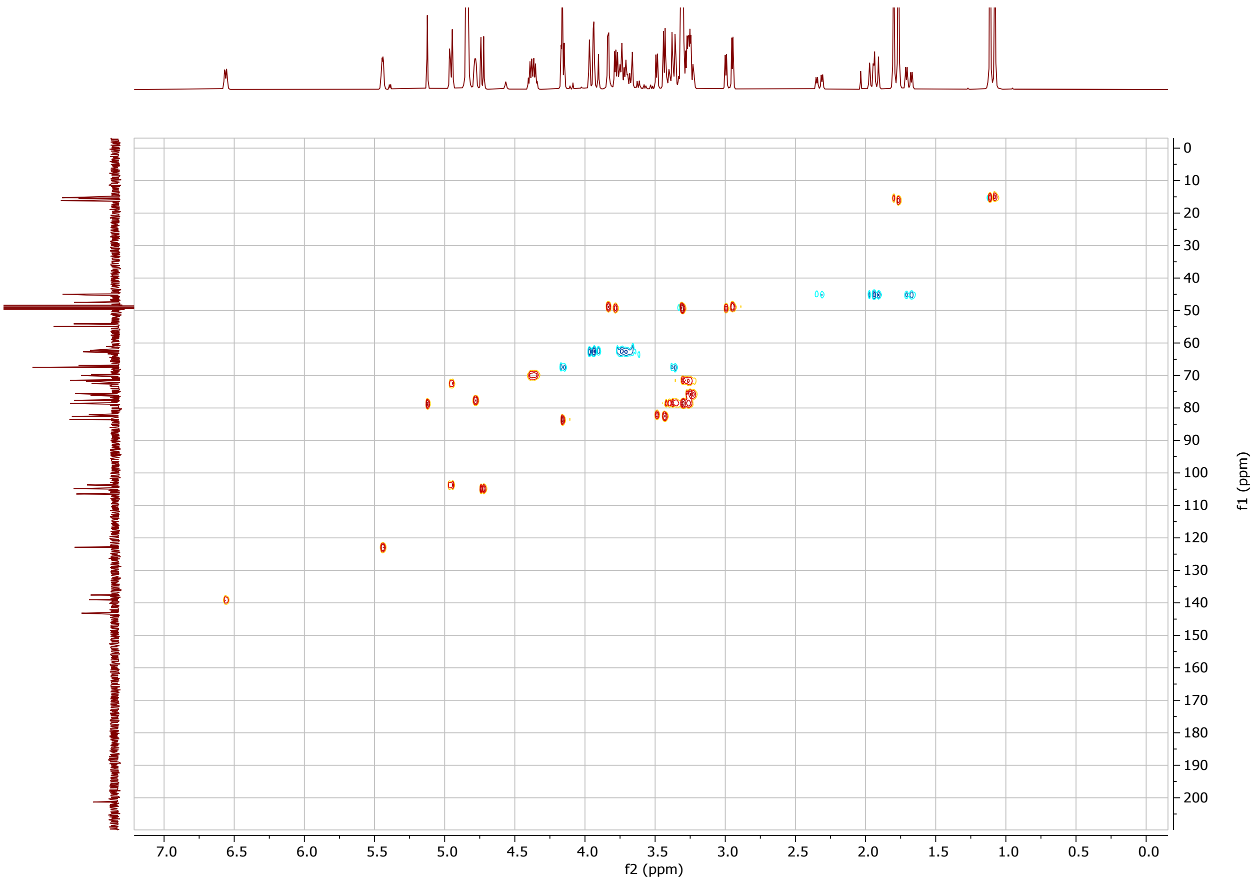

Figure S3. HSQC spectrum for the YjiC biotransformation product in CD_3OD

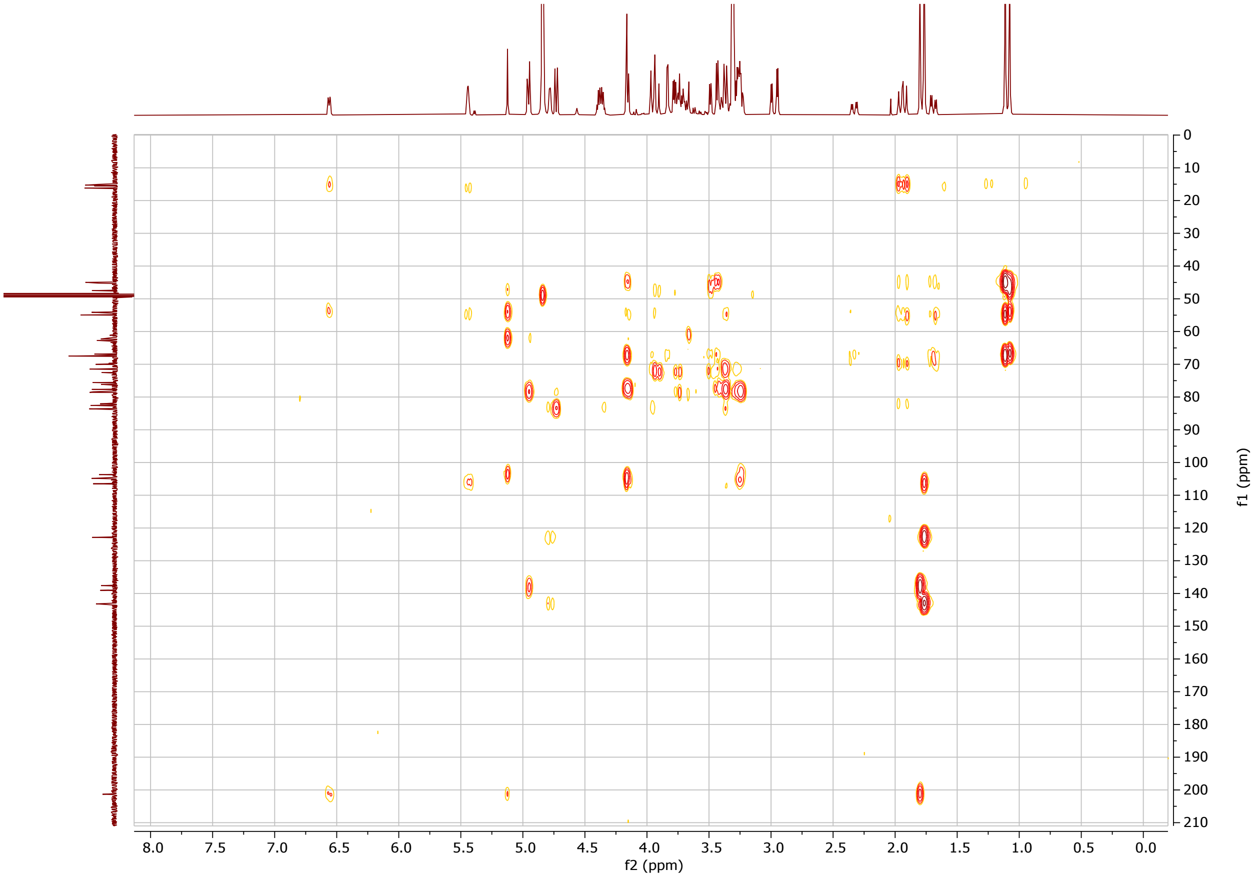

Figure S4. HMBC spectrum for the YjiC biotransformation product in CD_3OD

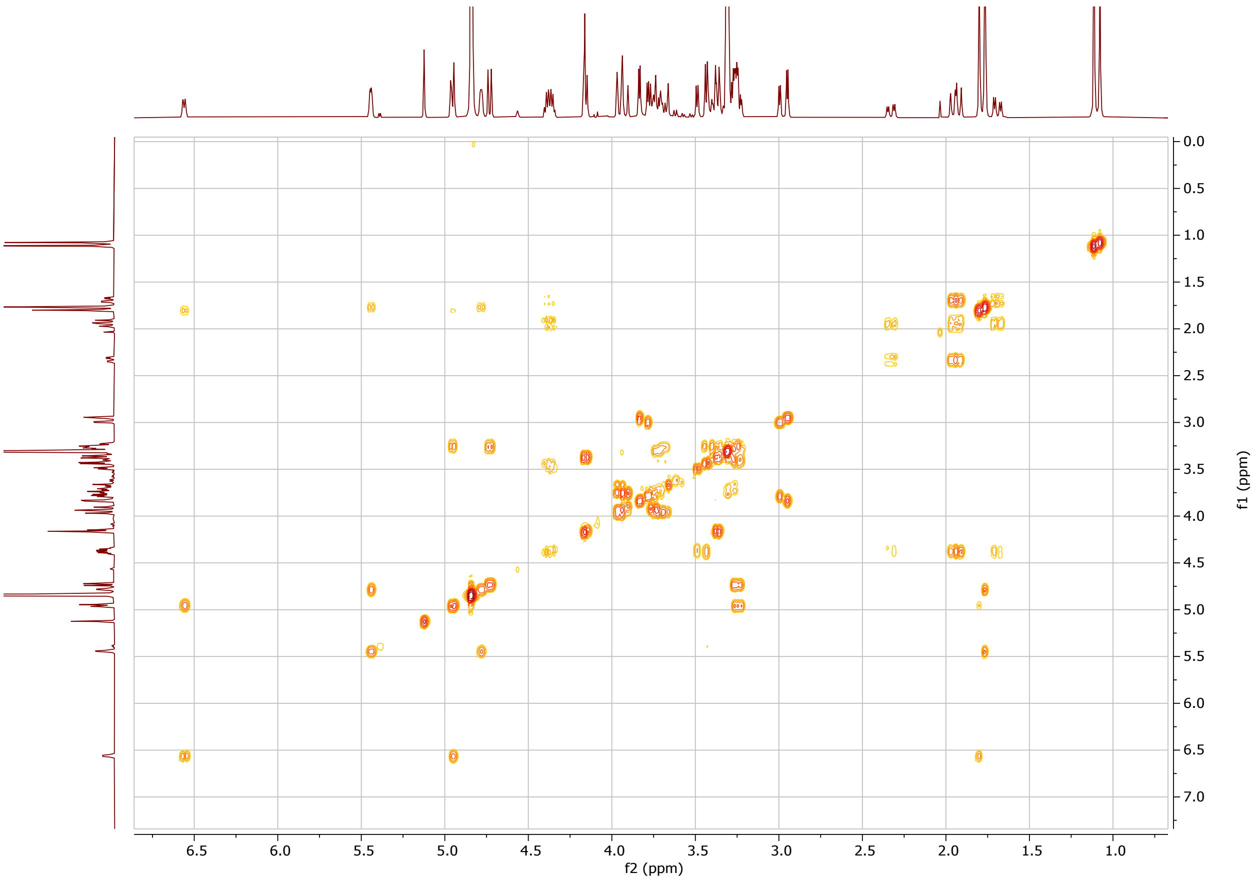

Figure S5. COSY spectrum for the YjiC biotransformation product in CD_3OD
